# Scattering asymmetry and circular dichroism in coupled PT-symmetric chiral nanoparticles

**DOI:** 10.1515/nanoph-2021-0705

**Published:** 2022-02-11

**Authors:** Xiaolin Chen, Hongfei Wang, Jensen Li, Kwok-yin Wong, Dangyuan Lei

**Affiliations:** State Key Laboratory of Chemical Biology and Drug Discovery, Department of Applied Biology and Chemical Technology, The Hong Kong Polytechnic University, Hong Kong 999077, China; Department of Optical Engineering, School of Physics, Hefei University of Technology, Feicui Road 420, Hefei 230601, China; Department of Materials Science and Engineering, City University of Hong Kong, Hong Kong 999077, China; Department of Physics, The Hong Kong University of Science and Technology, Clear Water Bay, Kowloon, Hong Kong, China

**Keywords:** chirality, circular dichroism, light scattering, parity-time symmetry, scattering matrix

## Abstract

We investigate the scattering properties of coupled parity-time (PT) symmetric chiral nanospheres with scattering matrix formalism. The exceptional points, i.e., spectral singularities at which the eigenvalues and eigenvectors simultaneously coalesce in the parameter space, of scattering matrix can be tailored by the chirality of the nanospheres. We also calculate the scattering, absorption and extinction cross sections of the PT-symmetric chiral scatter under illumination by monochromatic left- and right-circularly polarized plane waves. We find that the scattering cross section of the nanostructures exhibits an asymmetry when the plane waves are incident from the loss and gain regions, respectively, especially in the broken phase, and the optical cross section exhibits circular dichroism, i.e., differential extinction when the PT-symmetric scatter is endowed with chirality. In particular, under illumination by linearly polarized monochromatic plane waves without intrinsic chirality, the ellipticity of scattered fields in the forward direction, denoting the chirality of light, becomes larger when the scatter is in the PT-symmetry-broken phase. Our findings demonstrate that the gain and loss can control the optical chirality and enhance the chiroptical interactions and pave the way for studying the resonant chiral light–matter interactions in non-Hermitian photonics.

## 1 Introduction

Chirality, which refers to a geometric handedness of a chiral object that the mirror image cannot be superposed onto itself, is ubiquitous in nature, known for chiral molecules and the double-helix structure of DNA [1]. Analogously, light also exhibits chiral properties and enables carrying orbital angular momentum (OAM) and/or spin angular momentum (SAM), such as OAM-carrying vectorial light beams and SAM-carrying circularly polarized light [2]. Recently, the chiral light–matter interactions have attracted great interest in the optical community [3], and inspired technologically important applications such as chiral sensing [4]. Various nanostructures and relevant physical mechanisms have been designed and studied to enhance the chiroptical response, including the use of chiral plasmonic nanostructures [5], high-index dielectric nanostructures [6, 7], helicity-preserving optical cavities [8], planar dielectric nanostructures [9] and chiral metamaterials designed by deep learning [10].

Non-Hermiticity in many physical systems has drawn great attention in recent years since the discovery of real spectra in parity-time (PT) symmetric complex Hamiltonians [11], which commutes with PT operator, [H, PT] = 0, where the parity operator P amounts to reversal of spatial coordinates, r→−r , and the time-reversal operator T ( t→−t ) represents complex conjugation under steady states. Due to the formal equivalence between Maxwell and Schrodinger equations [12], optics has provided an ideal playground for the non-Hermitian physics in requesting the relative permittivity of an optical medium satisfies $\epsilon \left(r\right)=\epsilon^{*}\left(-r\right)$ , implying balanced loss and gain [13], because the optical gain and loss in photonic nanostructures can be controlled and integrated with high resolution, such as the ubiquitous dissipation due to the material absorption and radiation leakage, and optical gain induced through stimulated emission involving optical or electrical pumping [14]. As a result, a large number of intriguing non-Hermitian physical phenomena have been experimentally and theoretically demonstrated in photonics systems, typically including unidirectional invisibility [15], exceptional points (EPs) [14, 16], topological edge states [17], PT-coherent absorber and laser-absorber modes [18], [19], [20].

The light scattering of small particles or aggregates has been extensively studied in classical electrodynamics, such as the Mie scattering theory of optically active particles, due to various practical applications [21]. However, the research of combining chirality and PT symmetry in optical systems is rarely reported, except for a theoretical proposal of PT-symmetric chiral metamaterials consisting of slabs of opposite chirality [22, 23]. In this paper, we study the light scattering properties of a PT-symmetric chiral nanosphere dimer using the electromagnetic transition matrix (T-matrix) and scattering matrix (S-matrix) [18, 24]. By investigating the influence of chirality and non-Hermiticity on the extinction, scattering, absorption cross sections and ellipticity of scattered light of the dimer system, we reveal that the gain and loss play an important role in chiral optical systems that can enhance the chiroptical responses and also allow to manipulate exceptional points by the chirality. The interplay between PT-symmetry and chirality investigated in nanoparticle systems can also be potentially revealed through far-field scatterings.

## 2 Methods

### 2.1 T-matrix and S-matrix

**Figure 1:**
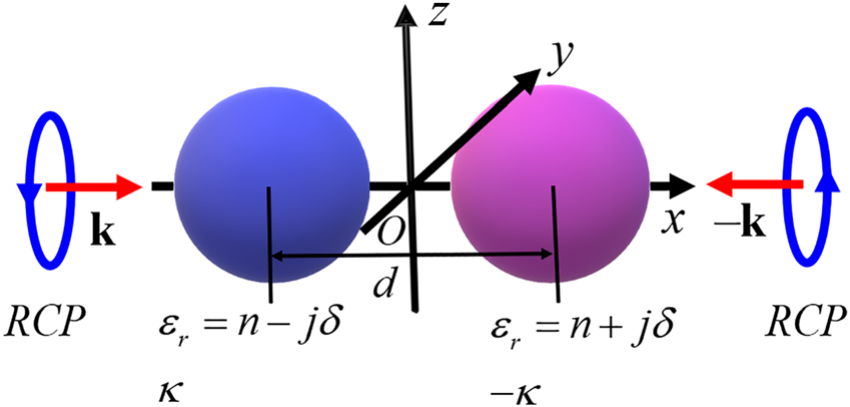
Two chiral nanoparticles positioned symmetrically along the *x*-axis to form a PT-symmetric optical scattering system. The distance between the centres of two spheres is *d*. The radii of two spheres are *a*, the relative permittivity of gain sphere is $\epsilon_{\text{r}}=n-j\delta$ with chirality parameter κ , the relative permittivity of loss sphere is $\epsilon_{\text{r}}=n+j\delta$ with chirality parameter −κ . The background environment is vacuum.

As sketched in Figure 1, we study the chiral electromagnetic response of a pair of chiral nanoparticles with the macroscopic Maxwell’s equations and constitutive equations (the time dependence ${\text{e}}^{-j\omega t}$ is assumed) [25], given by

(1) $$\begin{array}{l} D=\epsilon_{0}\epsilon_{\text{r}}E+j\kappa \sqrt{\mu_{0}\epsilon_{0}}H,\\ B=\mu_{0}\mu_{\text{r}}H-j\kappa \sqrt{\mu_{0}\epsilon_{0}}E, \end{array}$$

where $\epsilon_{0}$ and $\mu_{0}$ are the permittivity and permeability in vacuum, respectively. $\epsilon_{\text{r}}$ and $\mu_{\text{r}}$ are the relative permittivity and permeability, respectively. κ is the chirality (Pasteur) parameter of optically active medium expressing the magnetoelectric coupling. Applying Eq. (1) and Maxwell’ equations, we obtain the isotropic chiral media wave equation,

(2) $$\nabla^{2}E+\frac{\omega^{2}}{c^{2}}\left(\mu_{\text{r}}\epsilon_{\text{r}}-\kappa^{2}\right)E+2\frac{\omega }{c}\kappa \nabla \times E=0,$$

To solve the wave equation, we can use the helicity to split the electric field into left circularly polarized (LCP) wave and right circularly polarized wave (RCP) [26], i.e., $E=E_{+}+E_{-},$ where the $E_{+}$ (LCP) and $E_{-}$ (RCP) are the eigenstates of helicity operator with eigenvalues +1 and −1, respectively [27]:

(3) $$\Lambda =\frac{J\cdot P}{\left|P\right|}=\frac{\left(L+S\right)\cdot P}{\left|P\right|}=\frac{S\cdot P}{\left|P\right|}=\frac{\nabla \times }{k},$$

where J,L and S are the total angular momentum, OAM and SAM, respectively, i.e., the helicity is the projection of the total angular momentum onto the linear momentum P. Utilizing the wave equation and helicity operator, we can obtain the dispersion relations for the two circularly polarized waves, $k_{\pm }=\left(\omega /c\right)\left(\sqrt{\mu_{\text{r}}\epsilon_{\text{r}}}\pm \kappa \right)$ , which give rise to the refractive indices for the LCP and RCP waves and the mean refractive index, $m_{\text{L}}=\sqrt{\mu_{\text{r}}\epsilon_{\text{r}}}+\kappa ,m_{\text{R}}=\sqrt{\mu_{\text{r}}\epsilon_{\text{r}}}-\kappa ,m=\left(m_{\text{L}}+m_{\text{R}}\right)/2$ , respectively. Outside the scatterer (without chirality, i.e., κ=0 ), the electric field obeying Eq. (2) can be expanded in terms of spherical vector wave functions (SVWFs) [13],

(4) $$E\left(r,\omega \right)=\sum_{\mu }\left[a_{\mu }F_{\mu }^{\left(1\right)}\left(kr\right)+f_{\mu }F_{\mu }^{\left(3\right)}\left(kr\right)\right],$$

where the subscripts μ≡(τkl) denotes the mode, degree and order of SVWFs, respectively, e.g., $F_{1kl}^{\left(1,2,3\right)}\left(kr\right)=M_{kl}^{\left(1,2,3\right)}\left(kr\right)$ and $F_{2kl}^{\left(1,2,3\right)}\left(kr\right)=N_{kl}^{\left(1,2,3\right)}\left(kr\right)$ . The expansion coefficients of the incident and scattered waves are related by the T matrix,

(5) $$\left[f_{\mu }\right]=T\left[a_{\mu }\right].$$

Applying the electromagnetic boundary condition at the surface of the chiral sphere, we can obtain the T-matrix of each single chiral sphere [28, 29],


(6) $$\text{T}=\left[\begin{array}{ll} -\beta_{l} & -\gamma_{l}\\ -\gamma_{l} & -\alpha_{l} \end{array}\right].$$

The T-matrix is nondiagonal, exhibiting the mixing of the electric and magnetic multipolar modes due to the chirality, and the Mie coefficients are,

(7) $$\begin{array}{l} \beta_{l}=\frac{W_{l}\left(L\right)B_{l}\left(R\right)+W_{l}\left(R\right)B_{l}\left(L\right)}{W_{l}\left(L\right)V_{l}\left(R\right)+V_{l}\left(L\right)W_{l}\left(R\right)},\\ \alpha_{l}=\frac{V_{l}\left(R\right)A_{l}\left(L\right)+V_{l}\left(L\right)A_{l}\left(R\right)}{W_{l}\left(L\right)V_{l}\left(R\right)+V_{l}\left(L\right)W_{l}\left(R\right)},\\ \gamma_{l}=\frac{W_{l}\left(R\right)A_{l}\left(L\right)-W_{l}\left(L\right)A_{l}\left(R\right)}{W_{l}\left(L\right)V_{l}\left(R\right)+V_{l}\left(L\right)W_{l}\left(R\right)}, \end{array}$$

where $W_{l},V_{l},A_{l},B_{l}$ are given as [21],

(8) $$\begin{array}{l} W_{l}\left(J\right)=m\psi_{l}\left(m_{J}x\right){\xi^{\prime }}_{l}\left(x\right)-\xi_{l}\left(x\right){\psi^{\prime }}_{l}\left(m_{J}x\right),\\ V_{l}\left(J\right)=\psi_{l}\left(m_{J}x\right){\xi^{\prime }}_{l}\left(x\right)-m\xi_{l}\left(x\right){\psi^{\prime }}_{l}\left(m_{J}x\right),\\ A_{l}\left(J\right)=m\psi_{l}\left(m_{J}x\right){\psi^{\prime }}_{l}\left(x\right)-\psi_{l}\left(x\right){\psi^{\prime }}_{l}\left(m_{J}x\right),\\ B_{l}\left(J\right)=\psi_{l}\left(m_{J}x\right){\psi^{\prime }}_{l}\left(x\right)-m\psi_{l}\left(x\right){\psi^{\prime }}_{l}\left(m_{J}x\right), \end{array}$$

where J=L,R corresponding to LCP and RCP waves, respectively, the size parameter is x=ka , and $\psi_{l},\xi_{l}$ are Riccati–Bessel functions. When the chirality parameter is zero, $\gamma_{l}=0$ . The T-matrix for a cluster of spheres can be derived by the dyadic Green function [13]. For an arbitrary object, the T-matrix can be numerically calculated through the multiple plane wave illumination method [30] or the finite element method [31]. On the other hand, the electric field outside the scatterer can be purposely expanded by the incoming and outgoing channels using Hankel functions only:

(9) $$E\left(r,\omega \right)=\sum_{\mu }\left[\psi_{\mu }F_{\mu }^{\left(2\right)}\left(kr\right)+\varphi_{\mu }TF_{\mu }^{\left(2\right)}\left(kr\right)\right],$$

where $TF_{\mu }^{\left(2\right)}\left(kr\right)={F_{\mu }^{\left(2\right)}}^{\text{*}}\left(kr\right)$ showing that the outgoing channel is the time reversal of the incoming channel. The scattering matrix which relates the expansion coefficients of incoming and outgoing channels,

(10) $$\left[\varphi_{\mu }\right]=S\left[\psi_{\mu }\right],$$

can be derived from the T-matrix with the aid of relation between SVWFs [13]. To find the PT-symmetric conditions of the chiral scattering system, we take the action of PT operator on the wave equation, i.e., Eq. (2), and obtain,

(11) $$\kappa \left(r\right)=-\kappa^{*}\left(-r\right),\mu_{\text{r}}\left(r\right)=\mu_{\text{r}}^{*}\left(-r\right),\epsilon_{\text{r}}\left(r\right)=\epsilon_{r}^{*}\left(-r\right).$$

The scattering matrix of a general PT-symmetric optical system has the fundamental relation [18],

(12) $$\mathrm{PT}S\left(\omega^{*}\right)\mathrm{PT}=S^{-1}\left(\omega \right).$$

The weaker condition than unitarity implies the existence of spontaneous PT-symmetry breaking where the eigenvalues of scattering matrix are not unimodular, i.e., the eigenstate can exhibit amplification or dissipation. If the chiral media is reciprocal, the chirality parameter *κ* is a real number. It is worthy to note that the spontaneous PT-symmetry breaking of the scattering matrix indeed coincides with the PT-symmetry-breaking transition of the underlying PT-symmetric Hamiltonian [32].

### 2.2 Optical cross section

The scattering and absorption cross sections of the chiral optical system can be derived from the conservation of energy [33–35], when a monochromatic plane wave illuminates the scatter, and the total fields outside a sphere that circumscribes the scatter can be decomposed into incident and scattered waves, i.e., $E=E^{i}+E^{s},H=H^{i}+H^{s}$ . The scattering cross section can be given by

(13) Csca=12Ii∮Re{Es×Hs∗}⋅dS,

where the $I_{i}$ is the energy flux of the incident wave, the integral is performed on a spherical area enclosing the scatterer. In addition, the incident plane waves can be expanded via SVWFs [13],

(14) $$E^{i}={\hat{e}}_{i}e^{jk\cdot r}=\sum_{kl}{\left(-1\right)}^{k}j^{l}\frac{2l+1}{l\left(l+1\right)}\left[{\hat{e}}_{i}\cdot C_{-kl}\left(\hat{k}\right)M_{kl}^{\left(1\right)}\left(kr\right)-j{\hat{e}}_{i}\cdot B_{-kl}\left(\hat{k}\right)N_{kl}^{\left(1\right)}\left(kr\right)\right],$$

where ${\hat{e}}_{i}$ is the polarization of incident plane waves, and $C_{kl}\left(\hat{k}\right),B_{kl}\left(\hat{k}\right)$ are the vector spherical harmonics (VSHs). The expansion coefficients of incident plane waves are,

(15) $$\left[a_{\mu }\right]={\left(-1\right)}^{k}j^{l}\frac{2l+1}{l\left(l+1\right)}\left[{\hat{e}}_{i}\cdot C_{-kl}\left(\hat{k}\right),-j{\hat{e}}_{i}\cdot B_{-kl}\left(\hat{k}\right)\right],$$

According to the T-matrix, the scattering cross section can also be given by

(16) $$C_{\text{sca}}=\frac{1}{I_{i}}\frac{4\pi }{k^{2}}\frac{1}{2\eta }\sum_{kl}E_{kl}\left[{\left|f_{1kl}\right|}^{2}+{\left|f_{2kl}\right|}^{2}\right],$$

where $E_{kl}=\left[l\left(l+1\right)\left(l+k\right)!\right]/\left[\left(2l+1\right)\left(l-k\right)!\right],$ and η is the vacuum impedance. The absorption cross section can then be calculated by a volume integral on the scatterer by [7, 35]

(17) Cabs=12Ii∫Re{∂B∂t⋅H∗+∂D∂t⋅E∗}dV=ω2Ii∫[Im{μr}μ0|H|2+Im{ϵr}ϵ0|E|2]dV+ωcIi∫Im{κ}Im{H∗⋅E}dV.

It is obvious that the absorption in the chiral medium has three contributions, while the absorption due to the chirality parameter is proportional to the optical chirality [36],

(18) C=ω2c2Im{E⋅H∗}.

The extinction cross section is $C_{\text{ext}}=C_{\text{sca}}+C_{\text{abs}}$ . In addition, the extinction cross section can be derived from the T-matrix,

(19) Cext=−1Ii4πk212ηRe∑klEkl[a1kl∗f1kl+a2kl∗f2kl]

Note that the extinction and absorption cross sections of an optical scattering system can be negative in the presence of gain.

## 3 Results and discussions

We investigate the scattering properties of PT-symmetric coupled chiral spheres based on the theory and methods developed in the previous section. Our scattering system is depicted in Figure 1: two chiral nanoparticles both with a radius *a* = 60 nm positioned symmetrically along the *x*-axis, and their centre-to-centre distance is *d* = 130 nm. The sphere with optical gain is placed at −65 nm, and the lossy sphere is at 65 nm. The real part of relative permittivity is n=1.5 . To implement the realistic chiral sphere, one can coat a sphere made from a usual material with a shell composed of chiral organic molecules, helical nanostructures, or chiral metamaterials [37, 38]. In addition, it is feasible to realize dielectric gain nanoparticles by using PbS quantum dots doped glass and the relative permittivity can be adjusted through changing the diameter or the volume fraction of quantum dots in the glass matrix [39].

**Figure 2:**
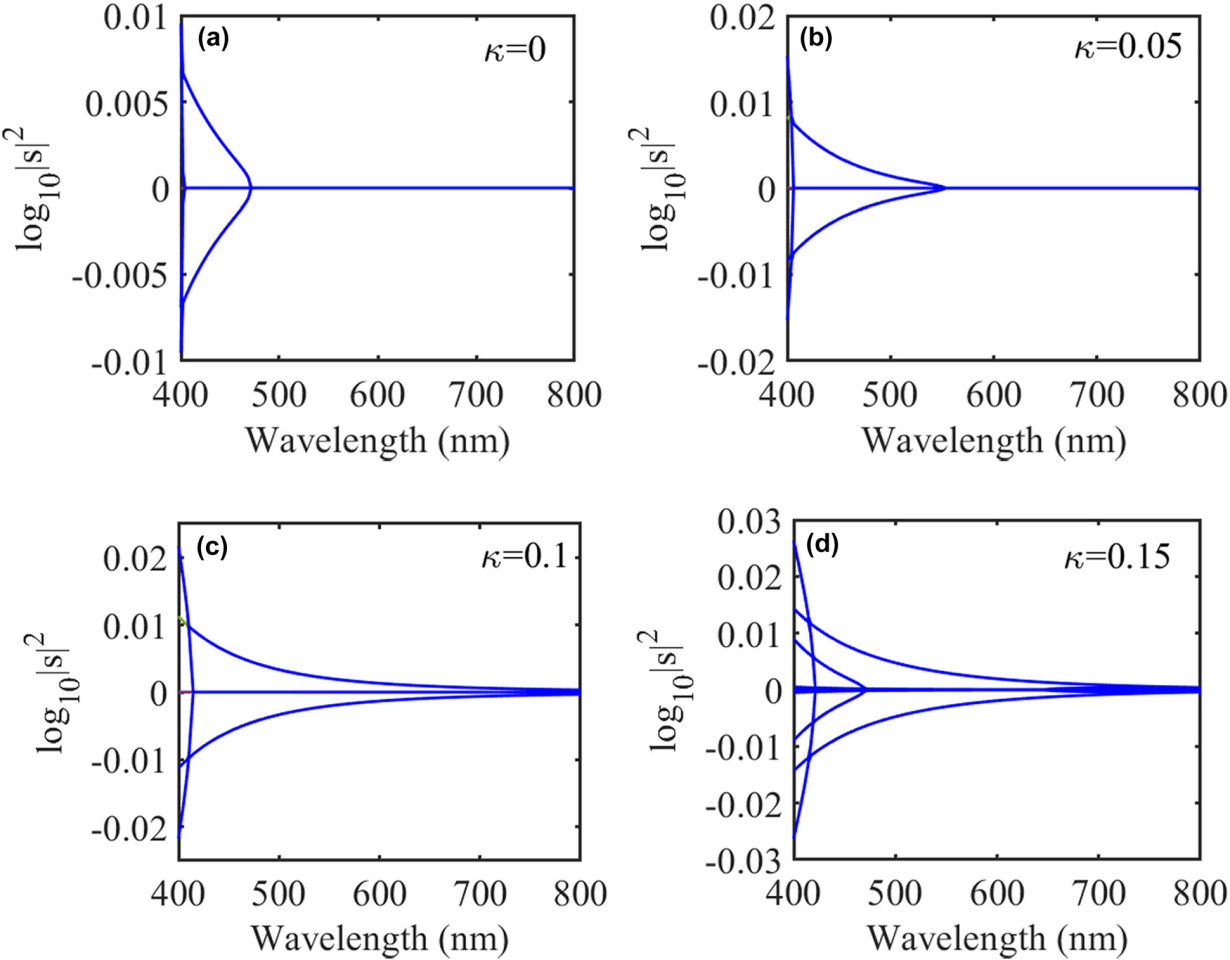
Semi-log plots of scattering matrix eigenvalues for the coupled PT-symmetric chiral nanospheres as a function of wavelength with chirality parameter κ=0 (a), κ=0.05 (b), κ=0.1 (c), and κ=0.15 (d). The non-Hermiticity parameter is δ=0.5 .

We first study the eigenvalues of the S-matrix of the nano-particle system influenced by the chirality parameter and only consider reciprocal chiral media, i.e., Im{κ}=0 [25]. The dimension of the S-matrix is 70 after truncating the scattered field expansions with a sufficient number of SVWFs. The eigenvalues as a function of wavelength *λ* varying from 400 to 800 nm are shown in Figure 2. When the chirality parameter is κ=0 , i.e., for a pair of PT-symmetric achiral nanoparticles, we can see that there exists a critical wavelength around $\lambda_{\text{EP}}=471$ nm, which denotes the onset of PT-symmetry breaking of the scattering matrix eigenstates. At the critical wavelength, the scattering matrix exhibits non-Hermitian degeneracies at which two scattering eigenstates and eigenvalues coalesce [32]. When the wavelength is larger than the critical wavelength, the S-matrix eigenvalues are all unimodular, i.e., $\left|s_{n}\right|=1$ . However, when the wavelength is smaller than the critical wavelength, one pair of eigenstates exhibit amplification and dissipation, respectively, with reciprocal moduli. When there is no gain and loss, the eigenvalues of the S-matrix are all unimodular.

Now we consider the chiral nanoparticles, when the chirality parameter is κ=0.05 , which is shown in Figure 2b, the critical wavelength red-shifts to $\lambda_{\text{EP}}=550$ nm. If we further increase the chirality parameter (Figure 2c and d), the critical wavelength is shifted beyond the wavelength range of interest and the PT-symmetric scattering system in the whole interested wavelength regime is in the PT-symmetry-broken phase. Therefore, the chirality parameter, i.e., the mixing of the electric and magnetic multipolar modes, plays an important role in the phase of PT-symmetric scattering systems. In the long-wavelength regime beyond the exceptional point, the loss and gain are unavoidably averaged out, resulting in the symmetric phase. The introduction of chirality, assisting the gain-loss contrast, pushes the exceptional point to a longer wavelength and provides another degree of freedom to tailor the exceptional points.

The optical cross section characterizes the capability of light concentration into sub-wavelength dimensions by metallic and dielectric nanoparticles. At low frequencies, the optical cross section of small nanoparticles is proportional to $\omega^{4}$ , i.e., the Rayleigh regime. The incident plane waves with different polarizations from the negative *x*-axis are,

(20) $$E^{i}=\frac{1}{\sqrt{2}}\left(e_{y}+\sigma je_{z}\right)\exp\left(jkx\right),$$

where σ=±1 corresponds to LCP and RCP plane waves, respectively. The opposite incident plane waves with same polarizations are related by the time-reversal operator. The optical chirality of the plane waves is given by

(21) $$C=\frac{\omega }{2c^{2}}\frac{1}{\eta }\sigma .$$

**Figure 3:**
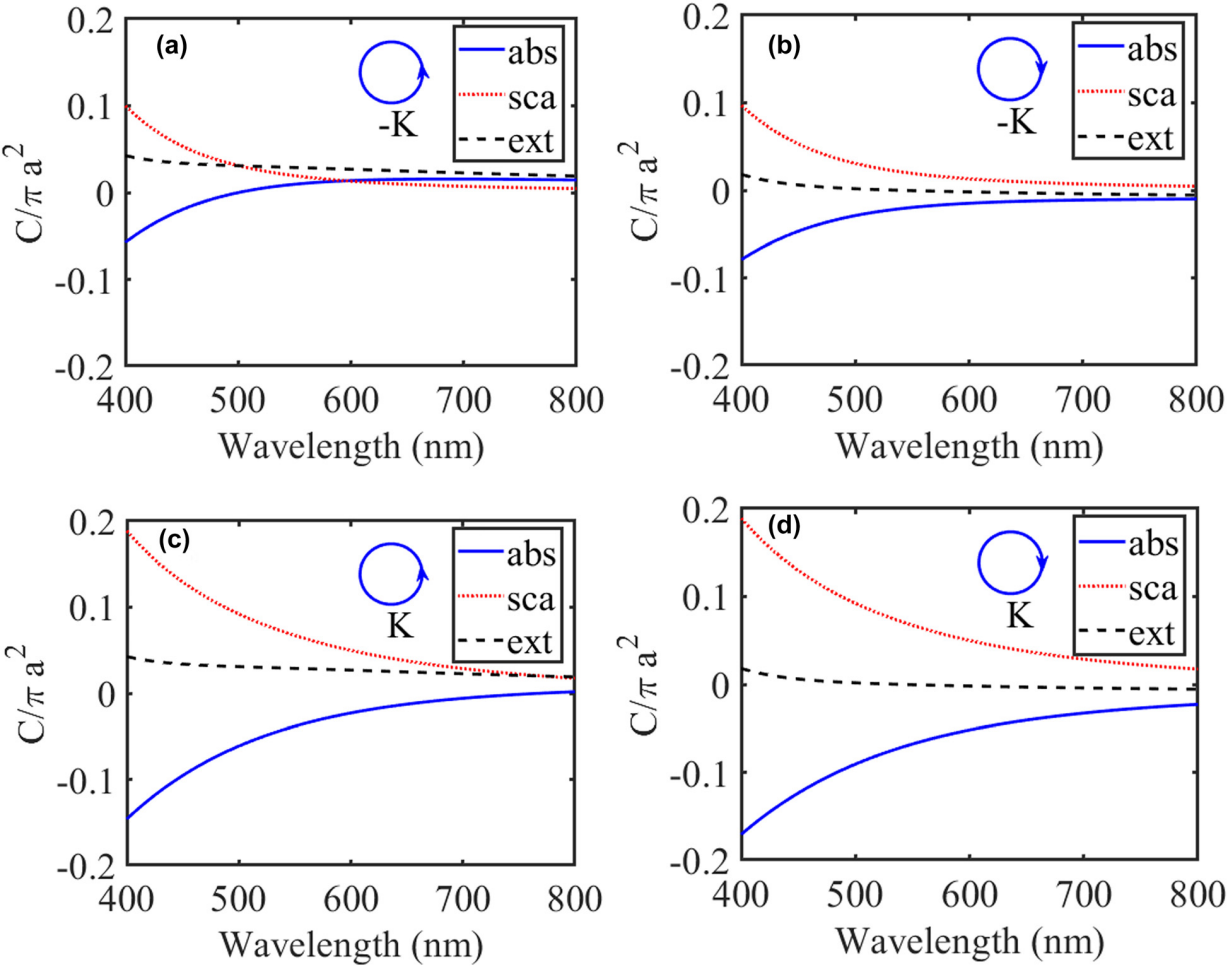
Normalized extinction (black dash lines), scattering (red dot lines), and absorption (blue lines) cross sections of the PT-symmetric chiral nanospheres. The left and RCP waves are incident from the lossy sphere (a) and (b) and from the gain sphere (c) and (d), respectively. The chirality parameter and non-Hermiticity parameter are κ=0.05 and δ=0.5 , respectively. The critical wavelength is $\lambda_{\text{EP}}=550$ nm.

The optical cross sections of the PT-symmetric chiral nanospheres are shown in Figure 3. Here, we set the chirality parameter and non-Hermiticity parameter as κ=0.05 and δ=0.5 , respectively. The absolute values of absorption cross section for LCP and RCP plane waves are small when the wavelength is larger than the critical wavelength $\lambda_{\text{EP}}=550$ nm. When the wavelength is smaller than the critical wavelength, the absorption cross section is negative and its absolute value becomes larger, which verifies that the PT-symmetric chiral scatter acts as an active particle. In addition, the absorption cross section is positive with wavelength varying from 500 to 800 nm when a LCP wave is incident on the sphere with optical loss. When the LCP and RCP waves are incident from opposite directions, the extinction cross sections are the same with the same polarization due to the Lorentz reciprocity [40],

(22) $$C_{\text{ext}}\left({\hat{e}}_{i},k\right)=C_{\text{ext}}\left({\hat{e}}_{i}^{*},-k\right).$$

While the extinction cross sections are different when the plane waves with opposite polarizations are incident from the same direction, so we can define the circular differential extinction cross section for chiral scatter, i.e., $CD_{\text{ext}}=C_{\text{ext,L}}-C_{\text{ext,R}}\neq 0$ . However, the scattering cross sections for opposite polarizations are approximately equal when the plane waves are incident from the same direction. But the scattering cross sections with the same polarization exhibit asymmetry when the plane waves are incident from opposite directions. It implies that we can design PT-symmetric chiral nanostructures to achieve unidirectional invisibility. Although there is no explicit transition in optical cross sections due to the excitation of multiple eigenstates under the plane wave illumination, a large scattering asymmetry is still observed in the PT-symmetry-broken phase.

**Figure 4:**
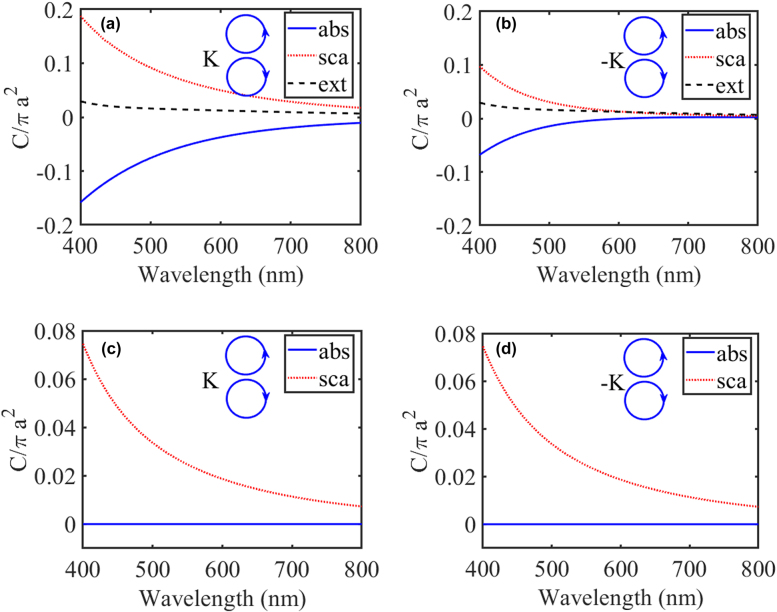
(a) Normalized extinction (black dash lines), scattering (red dot lines), and absorption (blue lines) cross sections of the PT-symmetric nanospheres without chirality, i.e., δ=0.5 and κ=0 (a) and (b), and chiral nanoparticles without gain and loss, i.e., δ=0 and κ=0.05 (c) and (d). The optical cross sections of LCP and RCP waves are the same when they are incident from the same directions.

To further demonstrate the scattering asymmetry, we calculate the optical cross sections of PT-symmetric nanospheres without chirality and coupled chiral nanoparticles without gain and loss, which are shown in Figure 4. In Figure 4a and b, we can see that the scattering asymmetry results from the gain and loss, but the extinction cross sections of LCP and RCP waves are the same when they are incident from same and reverse directions in the absence of chirality. When there is no gain and loss, the absorption cross sections of the coupled chiral nanoparticles are vanishing, so the scattering cross sections and extinction cross sections are equal. As a result of Lorentz reciprocity, the coupled chiral nanoparticles without gain and loss exhibit no scattering asymmetry. In addition, scattering cross sections are the same when the RCP and LCP waves are incident from the same directions, this can be explained by the total chirality of coupled nanospheres is zero.

**Figure 5:**
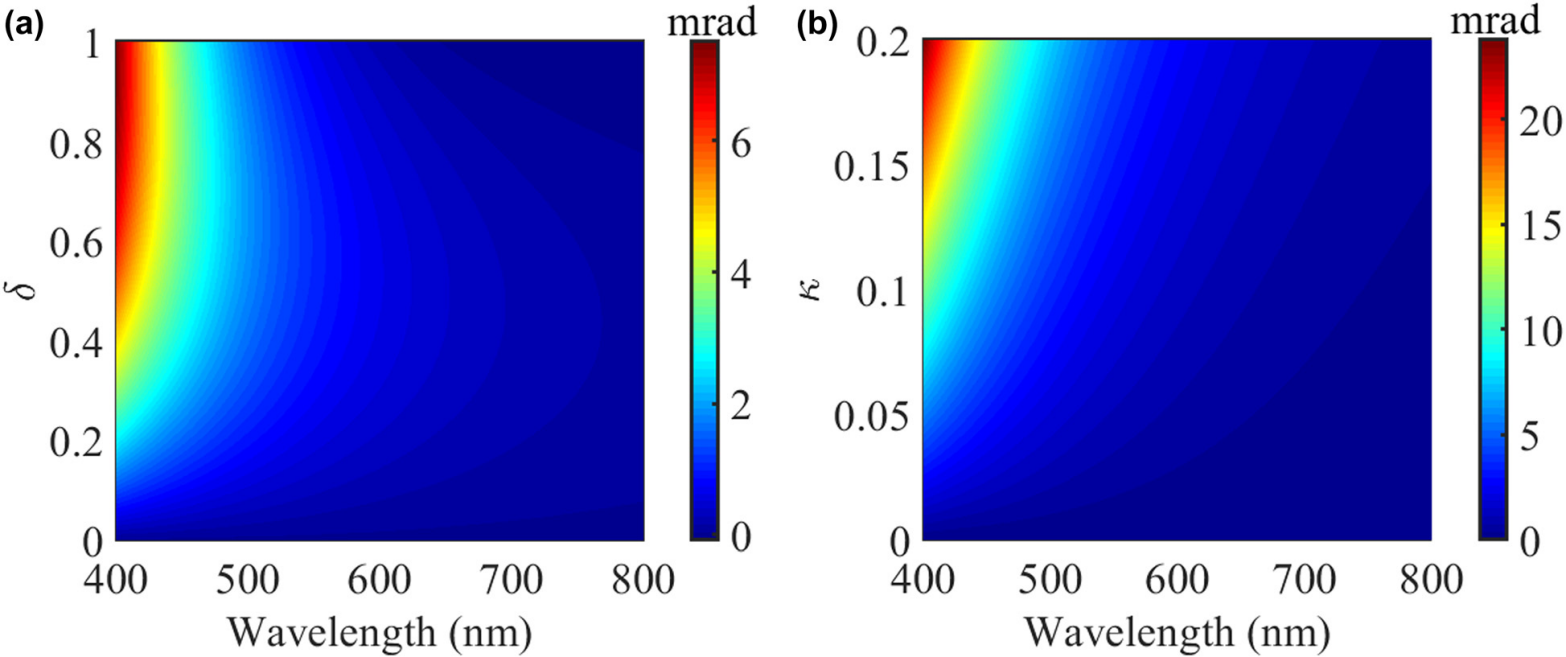
(a) Ellipticity of scattered fields in the forward direction as a function of non-Hermiticity parameter and wavelength. The chirality parameter is *κ* = 0.05. (b) Ellipticity of scattered field in the forward direction as a function of chirality parameter and wavelength. The non-Hermiticity parameter is *δ* = 0.5. The incident plane wave is linearly polarized plane wave without chirality.

Finally, we investigate the effect of PT-symmetry and chirality on the incident linearly polarized plane wave. The ellipticity of scattered fields is a widely used parameter to denote the polarization states of light and hence evaluate the chirality of an optical scattering system. For example, the ellipticities for right-handed circularly polarized waves, linearly polarized waves and left-handed circularly polarized waves are -*π*/4, 0 and *π*/4, respectively [21]. Since the light scattering amplitude in the forward direction is related to the extinction cross section according to the well-known optical theorem [41], we only need to calculate the ellipticities of scattered fields in the forward direction under linearly polarized plane wave illumination without chirality as shown in Figure 5. In Figure 5a, we can see that the ellipticities of scattered fields become greater than zero when the incident wavelength is smaller and the non-Hermiticity parameter becomes large. In other words, the chirality of scattered light becomes large when the scattering system is in the PT-symmetry-broken phase, while the scattered field in the forward direction exhibits no chirality in the unbroken phase. Likewise, In Figure 5b, when the non-Hermiticity parameter is *δ* = 0.5, the ellipticity of scattered fields becomes large when the scatter is in PT-symmetry broken phase when we tailor the chirality parameter.

## 4 Conclusions

We have studied the scattering properties of PT-symmetric chiral coupled spheres through electromagnetic T-matrix and S-matrix. We found that the exceptional points of S-matrix can be tailored by the chirality of nanospheres. When the PT-symmetric reciprocal chiral scatter is illuminated by the monochromatic LCP and RCP plane waves, the scattering and absorption cross sections exhibit asymmetry when the plane waves are incident from the loss and gain regions, respectively. While the scattering cross section are the same when the incident plane waves are incident from the same directions with different polarizations. In addition, the PT-symmetric reciprocal chiral scatter exhibits the circular dichroism, i.e., differential extinction. By calculating the ellipticity of scattered fields in the forward direction under linearly polarized monochromatic plane wave illumination, we also find that the ellipticity becomes larger when the scatter is in the PT-symmetric broken phase. Our results will provide help to study the enhanced chiroptical response by exploiting gain and loss in chiral nanostructures, such as the non-Hermitian topological chiral metamaterials.
